# Exploring *Castellaniella defragrans* Linalool (De)hydratase-Isomerase for Enzymatic Hydration of Alkenes

**DOI:** 10.3390/molecules24112092

**Published:** 2019-06-01

**Authors:** Matthias Engleder, Monika Müller, Iwona Kaluzna, Daniel Mink, Martin Schürmann, Erich Leitner, Harald Pichler, Anita Emmerstorfer-Augustin

**Affiliations:** 1 Austrian Centre of Industrial Biotechnology, Petersgasse 14, 8010 Graz, Austria; matthias.engleder@acib.at (M.E.); erich.leitner@tugraz.at (E.L.); harald.pichler@tugraz.at (H.P.); 2 Institute of Molecular Biotechnology, Graz University of Technology, NAWI Graz, BioTechMed Graz, Petersgasse 14, 8010 Graz, Austria; 3 InnoSyn B.V., Urmonderbaan 22, 6167 RD Geleen, The Netherlands; monika.muller@innosyn.com (M.M.); iwona.kaluzna@innosyn.com (I.K.); daniel.mink@innosyn.com (D.M.); martin.schuermann@innosyn.com (M.S.); 4 Institute of Analytical Chemistry and Food Chemistry, Graz University of Technology, NAWI Graz, Stremayrgasse 9, 8010 Graz, Austria

**Keywords:** hydratase, dehydratase, isomerase, monoterpene hydration, *Castellaniella defragrans*, myrcene, linalool, geraniol, tertiary alcohol

## Abstract

Acyclic monoterpenes constitute a large and highly abundant class of secondary plant metabolites and are, therefore, attractive low-cost raw materials for the chemical industry. To date, numerous biocatalysts for their transformation are known, giving access to highly sought-after monoterpenoids. In view of the high selectivity associated with many of these reactions, the demand for enzymes generating commercially important target molecules is unabated. Here, linalool (de)hydratase-isomerase (Ldi, EC 4.2.1.127) from *Castellaniella defragrans* was examined for the regio- and stereoselective hydration of the acyclic monoterpene β-myrcene to (*S*)-(+)-linalool. Expression of the native enzyme in *Escherichia coli* allowed for identification of bottlenecks limiting enzyme activity, which were investigated by mutating selected residues implied in enzyme assembly and function. Combining these analyses with the recently published 3D structures of Ldi highlighted the precisely coordinated reduction–oxidation state of two cysteine pairs in correct oligomeric assembly and the catalytic mechanism, respectively. Subcellular targeting studies upon fusion of Ldi to different signal sequences revealed the significance of periplasmic localization of the mature enzyme in the heterologous expression host. This study provides biochemical and mechanistic insight into the hydration of β-myrcene, a nonfunctionalized terpene, and emphasizes its potential for access to scarcely available but commercially interesting tertiary alcohols.

## 1. Introduction

**Scheme 1:**
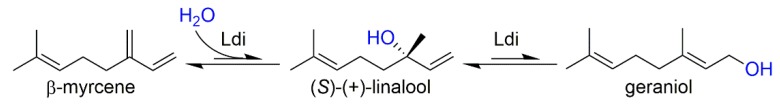
Ldi-catalyzed hydration of β-myrcene to (*S*)-(+)-linalool and its subsequent isomerization to geraniol.

Asymmetric addition of water to monoterpenes is of huge interest, since it enables access to high-value compounds from renewable, inexpensive starting materials [1]. The bifunctional linalool dehydratase-isomerase (Ldi, EC 4.2.1.127) offers access to the desired tertiary monoterpene alcohol (*S*)-(+)-linalool by reversible (de)hydration of β-myrcene (Scheme 1). (*S*)-(+)-linalool is isomerized to geraniol in a follow-up reaction catalyzed by the same enzyme [2]. The formation of (*S*)-(+)-linalool by Ldi is highly selective (e.e. ≥ 95%), and due to its poor commercial availability, the Ldi reaction may grant facilitated access to biotechnologically produced (*S*)-(+)-linalool [3,4].

Ldi catalyzes the initial steps of monoterpene mineralization in the facultative anaerobic β-proteobacterium *Castellaniella defragrans* strain 65Phen when grown under anaerobic conditions with β-myrcene as the sole carbon source [2]. In a proposed pathway, geraniol is then further metabolized to geranial and geranic acid by NAD^+^-dependent dehydrogenases, and introduced into β-oxidation. Since the thermodynamic equilibrium of the reactions favor isomerization of geraniol and dehydration of (*S*)-(+)-linalool, Ldi may additionally confer detoxification of monoterpene alcohols in vivo [2]. This was illustrated by 100- to even 1000-fold higher reaction rates for geraniol isomerization (V_max_ of approx. 25 µmol min^−1^ mg^−1^) and (*S*)-(+)-linalool dehydration (V_max_ of approx. 9 µmol min^−1^ mg^−1^) compared to the reverse reactions (V_max_ of approx. 8 nmol min^−1^ mg^−1^). Optimal conditions for the Ldi reaction were reported at pH 9.0 and 35 °C, and enzyme activity was inhibited when concentrations of NaCl, KCl, or MgCl_2_ exceeded 0.2 M. To date, the linalool isomerase from *Thauera linaloolentis* 47 Lol is the only sequence with notable similarity to Ldi [5], emphasizing the unique attributes of the enzyme in the protein sequence space.

**Figure 1:**
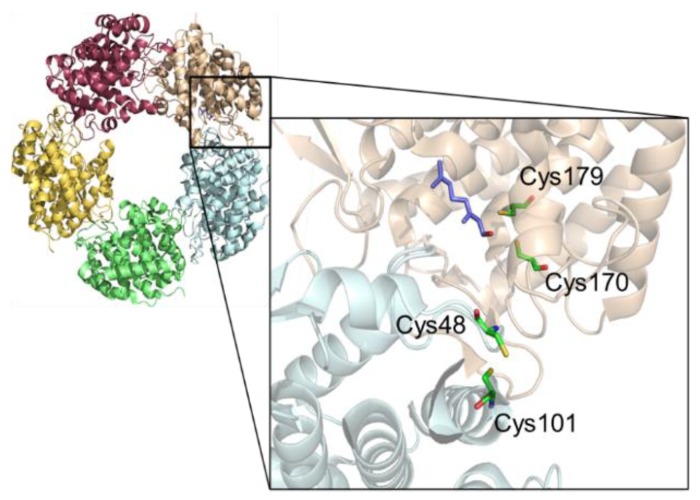
Overall structure and a close-up view of the active site of Ldi (PDB code: 5G1U [6]). Bound geraniol (purple) and the essential cysteines are highlighted in sticks. Cysteines 48 and 101 form a disulfide bond that caps off the active-site cavity. Reduced cysteines 170 and 179 in close vicinity to a monoterpenoid substrate participate in the catalytic mechanism.

Mature Ldi is a periplasmic protein. It is translocated to the periplasm via SEC-dependent membrane transport of the unfolded peptide mediated by an N-terminal signal sequence. Ldi is sensitive towards molecular oxygen and requires a mild reducing agent such as DTT for full activity in vitro. This suggests that the reduction–oxidation state of the four cysteines in the Ldi sequence is important for enzyme function [2,6]. While both eukaryotic and prokaryotic organisms keep their cytoplasm reduced which impairs disulfide formation, the oxidative environment in the bacterial periplasm allows for assembly of disulfide bonds [7]. In earlier work, Ldi was actively expressed in *E. coli* with the N-terminal secretion signal [6,8]. The crystal structure of Ldi from *C. defragrans* was independently solved in the groups of Harder as well as Grogan and Hauer. In both cases, the enzyme crystallized as toroidal pentamer, with each monomer showing an (α,α)_6_ barrel fold. The subunits, either obtained in complex with β-myrcene [6] or geraniol [6,8], are tightly joined by large interfacial areas. The active site of Ldi is located at the interface of two subunits, which is unique among (α,α)_6_ barrel proteins. The importance of cysteines for Ldi activity was highlighted by an essential disulfide bond capping the substrate channel and contribution of two additional cysteines in the putative reaction mechanisms (Figure 1) [6,8].

Both independent studies reported on acid/base catalysis for the (de)hydration and isomerization reactions of Ldi, in which a carbocation intermediate is formed from (*S*)-(+)-linalool via cysteine-catalyzed protonation, followed by rehydration for geraniol or deprotonation for β-myrcene [6,8]. Nestl and colleagues suggested a second mechanism that requires formation of a covalent thioterpene intermediate from (*S*)-(+)-linalool by attack of the terminal alkene by one of the active-site cysteines [8]. Investigation of the proposed mechanisms with combined quantum mechanics and molecular mechanics approaches revealed high relative Gibbs free energy barriers for the reaction pathways suggested by Nestl et al., which rendered them rather unfavorable [9]. In contrast, the calculations indicated that the catalytic reaction may instead be initiated by aspartic-acid-mediated protonation of C3′ of β-myrcene, giving an unstable carbocation intermediate that is subsequently hydrated by activated water to form (*S*)-(+)-linalool. This stable intermediate may then isomerize to geraniol by undergoing a concerted dehydration–hydration reaction conferred by an active-site cysteine and activated water, respectively [9].

In order to examine the biocatalytic potential of Ldi for hydration of monoterpenes, we compared several vectors and *E. coli* strains for functional expression of the enzyme and used both whole cells and isolated Ldi to stereoselectively convert β-myrcene to (*S*)-(+)-linalool. We investigated the role of periplasmic translocation by determining the assembly of subunits in our expression system and evaluated the function of the cysteines by site-directed mutagenesis. We found that a functional Ldi oligomer was formed only in the periplasm, while cytosolic expression resulted in a distinctly different assembly and complete loss of activity. Despite an unimpaired overall structure, this also applied for variants harboring single amino acid exchanges of essential cysteines, thus confirming the essential role of the thiols for Ldi structure and function.

## 2. Results and Discussion

### 2.1. Activity of Recombinant Ldi in E. coli

We initiated our investigations by evaluating different *E. coli* strains for recombinant expression of Ldi. We used an Ldi nucleotide sequence harboring the N-terminal, native signal sequence in codon optimized form, and compared different vector systems. The levels of recombinant protein in total cell lysates (TCLs) and cell-free extracts (CFEs) were assessed via SDS-PAGE and immunoblot analysis (Figure S2). Expression of Ldi varied notably among the tested strains and vectors. The amount of soluble Ldi obtained with *E. coli* BL21star (DE3), *E. coli* Origami, and *E. coli* LEMO21(DE3) harboring a pMS470 vector was too low for further use independent of the cultivation conditions, as the enzyme was mostly present in inclusion bodies. In consequence, we decided to evaluate several modified *E. coli* strains for potentially improved soluble expression of the enzyme (Table S7). Jointly, we exchanged the vector system from pMS470 to pET26b(+). This strategy led to vastly improved amounts of Ldi in CFEs (Figure S3). Due to the good yield of soluble enzyme as well as the relatively few additives needed for cultivation, we selected *E. coli* BL21-CodonPlus(DE3)-RP in combination with the pET26b(+) vector as the expression system for further work. In this setup, Ldi was expressed with its native N-terminal signal sequence and was fused to a C-terminal His_10_-tag. Most probably, the beneficial effect of this strategy for Ldi expression was a combination of the rather tightly regulated induction at a low concentration of 0.02 mM IPTG and the presence of extra copies of the *argU* and *proL* tRNA genes. This may favor translation of heterologous proteins from organisms with a GC-rich genome, such as *C. defragrans* (GC-content of 69%) [10,11]. Even though a codon-optimized version of Ldi was used in our studies, overall GC content was still slightly increased (57%).

**Figure 2:**
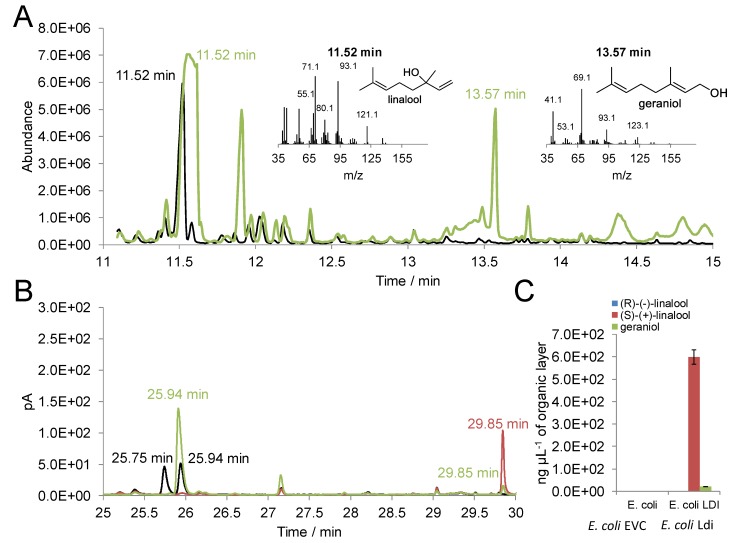
Whole-cell biotransformation of β-myrcene with *E. coli* expressing Ldi confirms enzyme activity. (**A**) GC-MS of an authentic standard of racemic linalool standard (black) and a representative sample from the biotransformation (green). (**B**) Chiral GC-FID of authentic racemic linalool (black) and geraniol (red) standards and a representative sample from the biotransformation (green). (*R*)-(−)-linalool and (*S*)-(+)-linalool were identified on basis of earlier publications [2,3] which showed that only the *S*-enantiomer of the tertiary alcohol is formed by Ldi. (**C**) Quantification of β-myrcene conversion to (*S*)-(+)-linalool and geraniol by an *E. coli* empty vector control (EVC) and *E. coli* cells expressing the enzyme (*E. coli* Ldi).

Focusing on the hydration reaction, functional expression of Ldi was confirmed by whole-cell biotransformations of β-myrcene (Figure 2). For unambiguous identification of reaction products from β-myrcene by GC–MS, the retention times and mass fragmentation patterns obtained from biotransformations were compared with an authentic standard of racemic linalool (Figure 2A). Only the conversions with *E. coli* overexpressing Ldi resulted in additional peaks absent from the control reactions with the empty vector control (EVC). Due to the formation of (*S*)-(+)-linalool and geraniol, Ldi-catalyzed hydration and isomerization of monoterpenoids as well as expression of the enzyme in its active form were confirmed. As reported previously [2,4], biotransformation of β-myrcene to (*S*)-(+)-linalool was highly stereoselective, since (*R*)-(−)-linalool was not detected by chiral GC (Figure 2B). In addition to the formation of approx. 600 ng µL^−1^ of (*S*)-(+)-linalool in the organic β-myrcene phase, small amounts of geraniol were generated during conversion (Figure 2C). This was also in accordance with the earlier observation that geraniol is formed only after accumulation of a certain concentration of (*S*)-(+)-linalool, as well as the supposed thermodynamic equilibrium of the Ldi reaction on the side of geraniol isomerization and (*S*)-(+)-linalool dehydration, respectively [2].

### 2.2. In Vitro Activity of Purified Ldi

**Figure 3:**
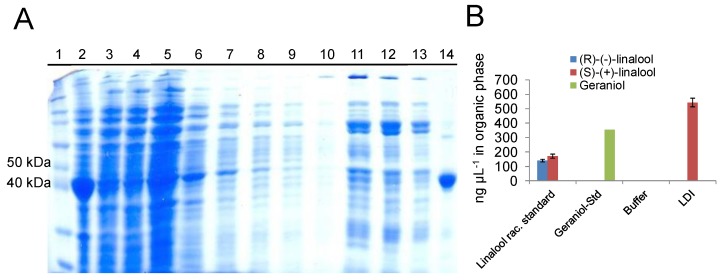
Purified Ldi catalyzes the enantioselective formation of (*S*)-(+)-linalool from β-myrcene. (**A**) Purification of Ldi from *E. coli* lysate via Ni-NTA affinity chromatography. Four µL of TCL and CFE (lanes 2 and 3), 10 µL of flow through (lane 4), washing fractions with 10 mM imidazole (lanes 5 to 10) and washing fractions with 50 mM imidazole (lanes 11 to 13), as well as 1 µL of purified and concentrated Ldi were loaded onto the gel. Novex^TM^ Sharp Prestained Protein Standard was used as the molecular weight marker (lane 1). (**B**) In vitro conversions of β-myrcene with 1 mg mL^−1^ of purified Ldi.

After achieving proper expression of soluble Ldi in *E. coli* and verification of activity, C-terminally His_10_-tagged Ldi was purified from *E. coli* lysate with self-packed Ni-NTA affinity chromatography columns (Figure 3A). Highly pure Ldi with an apparent molecular weight of approx. 45 kDa for the monomer was obtained via affinity tag purification. This matched well with the expected size of the mature protein without the native periplasmic targeting sequence (41.8 kDa). Ldi was most likely purified as a cofactor-free enzyme as indicated by the virtually colorless solution and the absence of any chromophores absorbing between 300 and 800 nm in the UV-Vis absorption spectrum (Figure S4). Directly after isolation, Ldi was subjected to activity tests with β-myrcene as the substrate (Figure 3B). Assays with purified Ldi under anaerobic conditions led to stereoselective formation of (*S*)-(+)-linalool. A clear difference between whole-cell and in vitro assays was the absence of detectable levels of geraniol with purified Ldi, which may have resulted from slightly lower (*S*)-(+)-linalool formation being prohibitive for the isomerization reaction [2].

### 2.3. Impact of Cysteine Exchange on Ldi Activity

Next, we focused on the role of the four cysteine residues in enzyme activity. We assessed whether mutating them to alanines and serines causes inactivation and/or disrupts the overall structure of the enzyme (Figure 1). We generated eight different single amino acid exchange variants of Ldi by site-directed mutagenesis and transformed *E. coli* BL21-CodonPlus(DE3)-RP with pET26b(+) plasmids harboring Ldi variants. Each variant was purified in parallel with Ldi wild type after confirming expression by immunoblot analysis (Figure S5A). We did not observe obvious differences between secondary structure elements of purified Ldi wild type and variant enzymes by CD spectroscopy, which indicated that none of the exchanges markedly affected the overall structure (Figure S5B). Consistent with previous data [6,7], site-directed mutagenesis of cysteines to alanines led to complete inactivation of the enzyme, underlining function of the thiols in active-site capping and catalysis. Earlier studies did not, however, investigate whether loss of Ldi activity was in fact a direct consequence of obstructing disulfide formation by cysteines 48 and 101 and acid/base catalysis aided by cysteines 170 and 179 [6,8], or rather caused by a disruption of the overall structure. Our results render the latter explanation highly unlikely and emphasize the involvement of oxidized and reduced thiols in active-site architecture and catalysis. Cysteine-supported acid/base catalysis is not unprecedented. For instance, catalytic cysteines have been described in maleate isomerase and glutamate racemase reactions [12,13]. We also created single amino acid exchange variants harboring serines instead of cysteines, and also ended up with properly folded but inactive enzyme (Table S8). For Ldi C48S and C101S, this was most likely due to elimination of the essential disulfide bond. In the case of Ldi C170S and C179S, we hypothesize that, in contrast to the thiols of the cysteines, the alcohol side chains of the serines will not be ionized at or near neutral pH. This would prevent formation of a covalently bound terpenoid intermediate resulting from nucleophilic attack on the substrate, and would object the proposed (de)hydration mechanism [8].

### 2.4. Periplasmic and Cytosolic Targeting of Ldi

**Figure 4:**
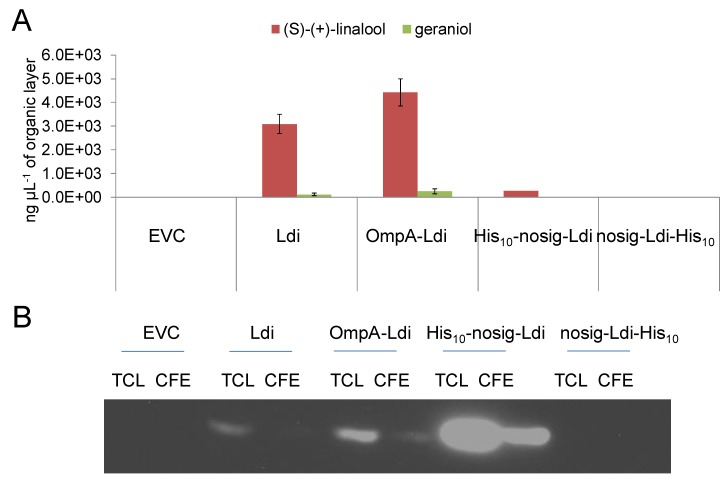
Subcellular targeting impacts the expression and activity of recombinant Ldi in *E. coli*. The enzyme was located to the periplasm by expressing Ldi constructs harboring either the native N-terminal signal sequence (Ldi) or an N-terminal *E. coli* OmpA signal sequence (OmpA-Ldi). Cytosolic enzyme localization was targeted with constructs lacking an N-terminal leader peptide (nosig-Ldi; either N- or C-terminally His_10_-tagged). (**A**) Whole-cell biotransformations of β-myrcene with *E. coli* expressing Ldi. (**B**) Expression analysis of Ldi in total cell lysates (TCL) and cell-free extracts (CFE) of an *E. coli* empty vector control (EVC), as well as in strains expressing Ldi, OmpA-Ldi, His_10_-nosig-Ldi, and nosig-Ldi-His_10_ by immunoblotting.

While our initial studies mainly addressed the expression and isolation of active Ldi in *E. coli*, we were still confronted with an overall yield of less than 1% of (*S*)-(+)-linalool from β-myrcene after 19 h of both in vitro and whole-cell reactions. Brodkorb and colleagues reported in their initial study on detection and isolation of Ldi from *C. defragrans* that the thermodynamic equilibrium of the reaction favors the formation of β-myrcene from (*S*)-(+)-linalool, which would strictly limit the hydration reaction [2]. Also, poor solubility of β-myrcene limits overall contact of enzyme and substrate, whereas solubility of (*S*)-(+)-linalool is better, which favors the dehydration reaction. However, several additional bottlenecks could limit β-myrcene hydration by Ldi. Supposing that either enzyme localization caused by periplasmic targeting, the limited expression level, or a combination of both were the most critical factors impacting the Ldi reaction, we expressed Ldi constructs with different N-terminal sequences in *E. coli* to investigate these assumptions in more detail. In addition to the C-terminally His_10_-tagged Ldi with its native signal sequence (Ldi), we designed an Ldi-His_10_-tag construct where the native N-terminal signal sequence was replaced by *E. coli* OmpA signal peptide [14] (OmpA-Ldi, Table S2) and tested whether this setup positively influenced the level of active protein in the periplasm. We also generated constructs lacking the N-terminal secretion signal but carrying a His_10_-tag either N- or C-terminally (His_10_-nosig-Ldi and nosig-Ldi-His_10_, Tables S3 and S4) to promote cytosolic Ldi localization and, thereby, avoid periplasmic translocation as a potential limitation for expression. The four different sequences were cloned into pET26b(+) and expressed in *E. coli* BL21-CodonPlus(DE3)-RP under the conditions described in the Materials and Methods section. Applying both N-terminal signal peptides, Ldi was predominantly translocated to the periplasm (Figure S6). Removing the signal sequence markedly affected protein secretion, since nosig-Ldi was mainly found in the cytosol. Comparing the hydration activity of different Ldi constructs in whole-cell biotransformations demonstrated a remarkable discrepancy between activity and expression level (Figure 4A,B).

Only Ldi constructs harboring an N-terminal signal peptide for periplasmic translocation were able to generate notable levels of (*S*)-(+)-linalool and small amounts of geraniol regardless of the low concentration of soluble protein (Figure 4B). OmpA-Ldi was expressed slightly better than Ldi, which correlated with a tendency for higher product yield. Ldi activity was severely affected when the enzyme was expressed without a signal peptide. C-terminally His_10_-tagged nosig-Ldi was not expressed at all and, consequently, no conversion of β-myrcene was observed. We were able to express nosig-Ldi by fusing an N-terminal His_10_-tag to the enzyme at an exceptionally high yield, but to our surprise, only ended up with traces of (*S*)-(+)-linalool. These results suggest several direct consequences of the Ldi signal sequence and its removal from the protein. By comparing His_10_-nosig-Ldi with nosig-Ldi-His_10_ we concluded an overall stabilizing effect of the N-terminal His_10_-tag for the protein. Equal conclusions were drawn in earlier studies for other proteins [15,16]. Since fusion of an N-terminal tag to Ldi or OmpA-Ldi would have resulted in masking the secretion signal for periplasmic localization, we refrained from expressing the corresponding Ldi constructs in *E. coli*. The vast disproportion between Ldi and His_10_-nosig-Ldi expression and activity indicated that the amount of soluble protein per se was not pivotal for activity. Instead, based on our data, we propose that the transport of the nascent polypeptide into the periplasm is much more decisive for Ldi function. The increased activity of the enzyme in the periplasm may be mainly linked to two factors. Firstly, Sec pathway-dependent translocation of Ldi [2] implies that the protein is translocated in an unfolded state and adopts the native overall fold only in the periplasm [17]. Considering that Ldi is a homopentamer in solution, this would be of particular importance for correct functional assembly of the oligomeric protein [6,8]. Secondly, as already discussed above, the Ldi 3D structure revealed that the reduction–oxidation state of its four cysteine residues needs to be strictly controlled for enzyme assembly and activity [6,8]. Oxidized cysteines 48 and 101 trigger formation of an intrasubunit disulfide bond for capping of the active-site cavity, while cysteines 170 and 179 must be kept in a reduced state for all Ldi reactions. Essentially, the formation and isomerization of disulfides in *E. coli* are only catalyzed in the periplasm by the Dsb protein family, which likely further accounts for the enhanced enzyme activity after periplasmic translocation [18,19].

### 2.5. Impact of Cellular Localization on Ldi Assembly and Activity

**Figure 5:**
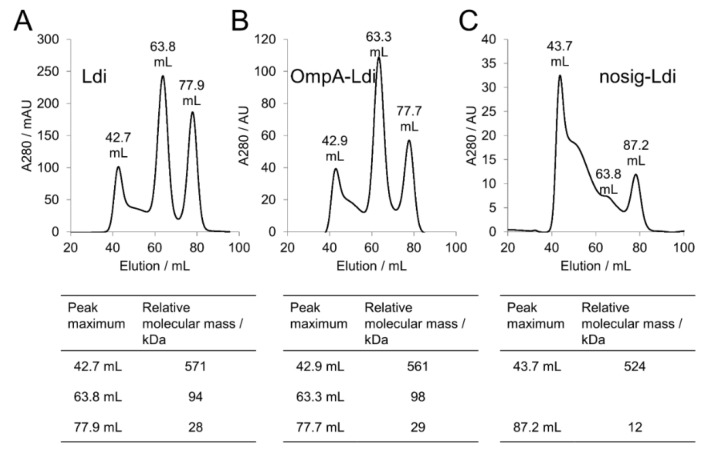
An N-terminal periplasmic signal sequence provokes formation of distinct Ldi oligomers in solution. Different oligomeric assemblies of His_10_-tag purified Ldi (**A**), OmpA-Ldi (**B**), and nosig-Ldi (**C**) in solution were separated by gel filtration. Relative molecular masses of major fractions were determined on basis of a calibration with standard proteins.

To examine the presumed central role of Ldi translocation on its correct functional assembly, we detected the oligomeric state of the different purified Ldi constructs via gel filtration. For Ldi and OmpA-Ldi, three major enzyme forms were obtained (Figure 5A,B). Elution times were largely independent of the Ldi construct investigated, except for one peak of the nosig-Ldi sample.

The peak with a maximum at approx. 43 mL was corresponding to an Ldi multimer, whereas the peaks with maxima at approx. 63 mL and 78 mL apparently corresponded to the elution of Ldi dimers and monomers, respectively. For both Ldi and OmpA-Ldi, the dimer was the most abundant form, followed by the monomer and the multimer. At approx. 54 mL, we expected to observe the peak maximum of an Ldi homopentamer—the native form of the protein in solution [6,8]. Instead, only a slight shoulder appeared at a roughly similar elution volume, indicating that the Ldi homopentamer was not one of the predominant protein forms in our expression system. Apart from the Ldi multimer, the elution profile of nosig-Ldi was characteristically different from the ones seen for Ldi and OmpA-Ldi (Figure 5C). Instead of Ldi dimers and monomers, we only encountered one additional distinct protein form with a peak maximum at 87.2 mL, and observed only a small shoulder instead of the dimer at approx. 64 mL. On the basis of our gel filtration calibration (Figure S1), we were not able to propose a conclusive enzyme assembly to these two fractions.

Upon activity tests with 1 mg mL^−1^ of separated fractions, Ldi and OmpA-Ldi dimers were the most active forms in solution (Table 1). The monomers showed a vastly reduced β-myrcene hydration activity (28% and 25% relative activity compared to Ldi and OmpA-Ldi dimers), and multimers were only barely active in the case of Ldi, and not active at all in the case of OmpA-Ldi. We attribute the apparent residual activity of the Ldi monomer fraction to an incomplete separation of the monomer and dimer in gel filtration. In consequence, this implies that the activity detected for the monomer was originating from residual Ldi dimer, whereas the monomer, in line with previous structural and biochemical investigations, was unable to catalyze the reaction. In vitro, all tested nosig-Ldi fractions were inactive. This was overall in good accordance with whole-cell biotransformations, where only a slight residual activity of nosig-Ldi was detected in spite of the notably higher expression level compared with Ldi and OmpA-Ldi (Figure 4).

Based on the fact that we detected enzyme activity in our assays even without obtaining the native pentameric structure, we propose that the minimum functional Ldi unit is a dimer. This is supported by the 3D structure, which suggests that two subunits would, in principle, be sufficient for (de)hydration and isomerization reactions at the interface of two monomers [6,8]. These results back our earlier hypothesis that oligomerization in the periplasm is eminent for the correct functional assembly of Ldi.

## 3. Conclusions

The highly enantioselective hydration of β-myrcene to (*S*)-(+)-linalool by Ldi permits production of a tertiary monoterpene alcohol that is only hardly accessible with other enzymes or organic biotransformations [3,4]. Characterization of this intriguing bifunctional (de)hydratase-isomerase has opened up routes towards its improvement, but also revealed challenges for its biocatalytic application. These can be primarily attributed to the low expression of functional Ldi and the low specific activity.

In this study, we tested functional expression of different Ldi constructs and amino acid exchange variants in *E. coli* and determined activities in whole-cell biotransformations and in vitro with isolated enzymes. Targeting studies demonstrated that a functional Ldi oligomer is assembled in the periplasm, and while the native enzyme in solution is a homopentamer, the minimal functional unit is a dimer. In addition to the appropriate functional assembly, periplasmic translocation is of utmost importance for formation of the correct reduction–oxidation state of each of the four essential cysteines. Periplasmic translocation is a common method for functional heterologous expression of disulfide-containing proteins in *E. coli*, but it is often connected with low yields due to yet poorly understood reasons [20]. In Ldi, this is aggravated by the heterogeneity of oligomers, which further reduces the efficiency of the *E. coli* expression system. The 1000-fold lower specific activity of Ldi for hydration of β-myrcene compared to the dehydration of (*S*)-(+)-linalool additionally hinders the commercial production of the more valuable monoterpene alcohol [2,21]. Further examination of whether Ldi is indeed limited by the thermodynamic equilibrium as hydration biocatalyst or can be developed by protein engineering or by modifying the reaction conditions to become a viable biocatalyst for the sustainable valorization of organic feedstock will follow.

## 4. Materials and Methods

### 4.1. Chemicals and Media

Unless otherwise stated, all chemicals, solvents, and reference compounds were obtained from Sigma-Aldrich^®^ (Steinheim, Germany) or Carl Roth GmbH & Co. KG (Karlsruhe, Germany) with the highest purity available. β-myrcene (purity ≥ 95%), racemic linalool (purity ≥ 97%), (*R*)-(−)-linalool (purity ≥ 99%), and geraniol (purity ≥ 99%) were purchased from Sigma-Aldrich^®^ (Steinheim, Germany). pEHISTEV [22] and pMS470 [23] vectors were employed for expression. Restriction enzymes were acquired from Thermo Scientific (St. Leon-Rot, Germany). Sterile water was purchased from Fresenius Kabi (Graz, Austria). For cloning and plasmid replication, *E. coli* Top10 F′ (F′[*lac*I^q^ Tn*10*(tet^R^)] *mcrA* Δ(*mrr*-*hsd*RMS-*mcrBC*) ϕ80*lacZ*ΔM15 Δ*lacX*74 *deoR nupG recA1 araD139* Δ(*ara-leu*)7697 *galU galK rpsL*(Str^R^) *endA1 λ*^−^) from Life technologies (Vienna, Austria) was used. Recombinant Ldi was expressed in *E. coli* BL21-CodonPlus(DE3)-RP (F-*ompT hsdS*(r_B_^−^ m_B_^−^) *dcm*^+^ Tet^r^ *gal endA* Hte [*argU proL* Cam^r^]) (Life technologies, Vienna, Austria).

### 4.2. Cloning and Site-Directed Mutagenesis

Standard molecular cloning technologies were used according to established procedures [24]. Correct integration of the insert was confirmed by sequencing (LGC Genomics, Berlin, Germany). For gene amplification, Phusion^®^ High Fidelity DNA polymerase (Thermo Fisher Scientific Inc., St. Leon-Rot, Germany) was utilized in accordance with the recommended PCR protocol. Codon-optimized gene variants of Ldi (GenBank: CBW30776.1) harboring the native N-terminal signal sequence (Ldi, Table S1) or, instead, an N-terminal *E. coli* OmpA signal sequence (OmpA-Ldi, Table S2) were obtained from DSM Ahead R & D (Geleen, The Netherlands). N- and C-terminally His_10_-tagged Ldi constructs without an N-terminal signal sequence (nosig-Ldi, Tables S3 and S4) were generated by PCR from an Ldi template using suitable primers with *Nde*I and *Hind*III restriction sites for cloning into pET-26b(+). Synthetic His_10_-tagged genes were amplified using primers with *Nde*I and *Hind*III restriction sites (Table S5) and were cloned into a pET-26b(+) expression vector (Merck, Germany). The resulting constructs were used to transform *E. coli* BL21-CodonPlus(DE3)-RP.

Single amino acid exchanges of cysteines to alanines or serines were introduced into the Ldi coding sequence by site-directed mutagenesis (Table S6). The Stratagene QuikChange site-directed mutagenesis protocol was slightly modified by preparing two separate PCR reactions containing forward and reverse primers, respectively. After five cycling steps, PCR reactions were combined and PCR was continued for 20 additional cycles according to the Stratagene manual. Mutated plasmids were verified by DNA sequencing of the coding regions of the constructs.

### 4.3. Expression of Recombinant Ldi in E. coli

Ldi constructs were expressed in *E. coli* BL21-CodonPlus(DE3)-RP. First, a preculture was inoculated in 10 mL LB supplemented with 15 µg mL^−1^ of kanamycin and 20 µg mL^−1^ of chloramphenicol and was cultivated at 25 °C and 130 rpm overnight. Main cultures were inoculated in LB to an OD_600_ of 0.1 and protein expression was induced with 0.02 mM IPTG at an OD_600_ of 0.6–0.8. Recombinant Ldi was expressed for 14 h at 25 °C and 130 rpm. Cells were harvested by centrifugation for 15 min at 4400× *g* and 22 °C and were used instantly for whole-cell biotransformations or were stored at −20 °C until protein purification.

### 4.4. Whole-Cell Biotransformation of β-myrcene with E. coli

Bioconversions of β-myrcene with *E. coli* were initiated immediately after protein expression. Fifty OD_600_ units, which correspond to a cell dry weight (CDW) of 50 mg, were harvested and resuspended in 1 mL of 20 mM sodium phosphate, pH 7.4, in Pyrex^®^ glass culture tubes. To initiate the reactions, 100 µL of β-myrcene were added to the cell suspensions. Biotransformations were incubated in technical triplicates for 19 h at 35 °C and 150 rpm with the reaction tubes fixed in a 55° angle relative to horizontal axis. Conversions were quenched by addition of 500 µL of ethyl acetate and monoterpenoids were extracted for 30 min at 1500 rpm on a Vibrax VXR basic (IKA, Germany). After phase separation by spinning for 5 min at 2900× *g* and 22 °C, terpenoids in 10 µL of organic phase were verified by GC-MS and were quantified by solid-phase microextraction (SPME) coupled to GC-FID.

### 4.5. Cell Lysis and Purification of Ldi

In order to purify recombinant Ldi from *E. coli*, cell pellets were resuspended in 50 mM sodium phosphate, pH 8.0, containing 10 mM imidazole and disrupted by ultrasonication for 4 min with a Sonifier^®^ 250 (Branson, Danbury, CT) at 80% duty cycle and output control 8. Cell-free extract (CFE) was separated from the total cell lysate (TCL) by centrifugation for 35 min at 48,300× *g* and 4 °C and was filtered through 0.22 µm filters (Millipore, Bedford, MA) prior to loading onto a pre-equilibrated self-packed Ni-NTA affinity chromatography column (GE Healthcare, Little Chalfont, United Kingdom). Nonspecifically bound protein was removed by washing the column with 7 column volumes of 50 mM sodium phosphate, pH 8.0, containing 10 mM imidazole and 5 column volumes of the same buffer containing 50 mM imidazole. His_10_-tagged Ldi was eluted with 7 column volumes of the same buffer containing 250 mM imidazole. The eluate was concentrated and buffer was exchanged to 80 mM Tris-HCl, pH 9.0, using disposable PD-10 desalting columns (GE Healthcare, United Kingdom) according to the recommended protocol.

Different oligomeric forms of Ldi were separated by gel filtration on a Superdex 200 HiLoad 16/60 column (GE Healthcare, United Kingdom) using 80 mM Tris-HCl, pH 9.0, for elution. The apparent relative molecular mass of native Ldi was determined by comparison of the size of eluted protein with a calibration curve (Figure S1) using γ-globulin (158 kDa), bovine serum albumin (66 kDa), ovalbumin (44 kDa), myoglobin (17 kDa), and Vitamin B_12_ (1.35 kDa) from a commercially obtained gel filtration standard set (Bio-Rad, Hercules, CA).

### 4.6. In Vitro β-myrcene Conversion

In vitro bioconversions were performed with purified Ldi. Before reactions, oxygen was removed from protein solutions by incubation for 1 h in a glove box under anaerobic conditions. Degassed Ldi was treated with either a 50-fold molar excess of DTT for 30 min or the same volume of ddH_2_O in controls. For conversions, 200 µL of β-myrcene were added to 400 µL of 1 mg mL^−1^ Ldi in Pyrex^®^ glass culture tubes. In vitro reactions were performed in technical triplicates for 19 h at 35 °C and 300 rpm in reaction tubes fixed in a 55° angle in a glove box for maintaining anaerobic reaction conditions. Conversions were stopped, extracted, and prepared for SPME coupled to GC-FID as described for the whole-cell biotransformations.

### 4.7. Analysis of Monoterpenoids by GC-MS and GC-FID

Monoterpenoids in organic solvent were identified by comparison of retention times and mass fragmentation patterns of analytes to authentic standards in gas chromatography–mass spectrometry (GC-MS). An HP-5 column (crosslinked 5% Ph-Me Siloxane; 30 m length, 0.25 mm in diameter, and 0.25 µm film thickness) on a Hewlett-Packard 6890 Series II GC equipped with a 5973 mass selective detector was used. Sample aliquots of 1 µL were injected in split mode (split ratio 5:1) at 230 °C injector temperature and 280 °C interface temperature with He as carrier at a flow rate set to 0.93 mL min^−1^ (35 cm/s linear velocity) in constant flow mode. The oven temperature program was as follows: 50 °C for 1 min, a ramp to 160 °C at 10 °C min^−1^, followed by a ramp to 250 °C at 20 °C min^−1^ (1.5 min). The total run time was 18 min. The mass selective detector was operated in a mass range of 35–300 amu at an electron multiplier voltage of 1671 V with a scan rate of 3.6 scans per second.

For separation of linalool enantiomers and quantification of monoterpenoids, a GC-FID method preceded by SPME for extraction of volatiles was developed. Therefore, a chiral BGB-174 column (30% 2,3-diacetyl-6-tert-butyLdimethylsilyl-beta-cyclodextrin dissolved in BGB-1701 (14% cyanopropylphenyl-, 86% methylpolysiloxane)) on a Hewlett-Packard 6890 GC equipped with a flame ionization detector (FID) was used. An automated SPME system was used for extraction and enrichment of the analytes on a divinylbenzene-carboxen-polydimethylsiloxane fiber (50/30 µm, 2 cm stable flex, Supelco, Bellfonte, PA, USA). A CTC Combi PAL sampler (CTC Analytics, Switzerland) equipped with a single magnetic mixer (Chromtech, Germany) was used for sampling. Samples were equilibrated in the autosampler for 5 min at 60 °C under stirring with a glass-coated magnetic stirrer. Extraction onto the SPME fiber was performed for 10 min at 60 °C. Then, the fiber was transferred to the GC injector for application of the sample onto the GC column. The oven program was as follows: 50 °C for 1 min and 6 °C min^−1^ ramp to 240 °C. The total run time was 36 min.

### 4.8. Protein Analysis and Immunoblotting

Routinely, Ldi expression levels in *E. coli* cell lysate and the levels of purified Ldi were assessed by SDS-PAGE or Western blot. SDS-PAGE was performed with the NuPAGE^®^ SDS-PAGE Gel System (Life Technologies, Austria) according to the manufacturer’s recommendations. For analysis of proteins via immunoblot, a primary anti-His antibody as well as an HRP-conjugated secondary antibody and enhanced chemiluminescent signal detection (SuperSignal^TM^, Pierce Biotechnology, Rockford, IL, USA) were used for visualization of immunoreactive bands.

### 4.9. Isolation of Recombinant Ldi from E. coli Periplasm and Cytosol

*E. coli* periplasm containing recombinant Ldi was isolated by preparing spheroplasts as described previously [25]. Briefly, after protein expression, cells were harvested for 4 min at 10,000× *g* and were resuspended in an equal volume of 10 mM Tris-HCl, pH 8.0. After centrifugation for 4 min at 10,000× *g*, cells were resuspended in an equal volume of spheroplasting buffer containing 20% sucrose and 10% of 0.1 M EDTA in 30 mM Tris-HCl, pH 8.0. A freshly prepared lysozyme solution was added to a concentration of 0.1 mg mL^−1^. The suspension was incubated for 30 min at 0 °C before separation of periplasm from spheroplasts by spinning for 10 min at 10,000× *g* and 4 °C. After withdrawing the supernatant containing periplasmic Ldi, the cytosolic Ldi fraction was recovered by disrupting spheroplasts with the B-PER^TM^ Bacterial Protein Extraction Reagent (Thermo Scientific, Germany) according to the recommended protocol. Periplasmic and cytosolic Ldi was analyzed by immunoblot analysis.

### 4.10. Circular Dichroism Spectroscopy

Circular dichroism (CD) spectral measurements of Ldi wild-type enzyme and single amino acid exchange variants were carried out with a Jasco J-810 spectropolarimeter (Jasco, MD). Far-UV CD spectra were collected between 190 nm and 260 nm with a scan speed of 50 nm min^−1^ in quartz cells of 5 mm path length at 22 °C. Protein concentrations were ranging from 6.3 µM to 13.4 µM in 80 mM Tris-HCl, pH 8.0. Obtained values were normalized by subtracting the baseline recorded for the buffer. Data were expressed in mean residue ellipticity [Θ] in deg cm^2^ dmol^−1^.

## Figures and Tables

**Table 1:** The oligomeric assembly of Ldi subunits in solution strongly affects enzyme activity. Ldi gel filtration fractions corresponding to the different oligomers in solution were pooled and applied in β-myrcene conversions for 19 h under anaerobic conditions. For each Ldi construct, the activities of different assemblies were set relative to the oligomeric form with the highest β-myrcene conversion. Pooled enzyme fractions without any hydration activity are denoted as ‘ia’ (inactive).

Ldi Construct	Pooled Fraction	Relative Molecular Mass/kDa	Assembly in Solution	Relative Activity for β-myrcene Hydration [%]
Ldi	37–46 mL	930–430	Multimer (>8 subunits)	4
	57–70 mL	168–55	Oligomer (2 to 4 subunints)	100
	72–84 mL	47–17	Monomer	28
OmpA-Ldi	38–47 mL	854–395	Multimer (>8 subunits)	ia
	58–70 mL	154–55	Oligomer (2 to 4 subunints)	100
	73–83 mL	43–18	Monomer	25
nosig-Ldi	40–48 mL	720–363	Multimer (>8 subunits)	ia
	60–70 mL	130–55	Inconclusive	ia
	74–83 mL	39–18	Inconclusive	ia

## Supplementary Materials

The following are available online, Figure S1: Gel filtration calibration curve generated with standard proteins listed in the Materials and Methods section; Figure S2: Initial expression studies of Ldi in different *E. coli* strains indicated poor expression of soluble protein. Ldi was expressed from a pMS470 vector with and without co-expression of DsbC. The level of Ldi in *E. coli* lysate was analyzed by immunoblotting; Figure S3: Expression of Ldi in different *E. coli* strains increases the yield of soluble protein in specific set ups. Nine different *E. coli* strains were tested for expression of Ldi, and after harvesting the cells, His_10_-tagged Ldi was detected in total cell lysates and cell free extracts by immunoblotting; Figure S4: UV-Vis absorption spectrum of purified Ldi. The enzyme was His_10_-tag purified from cell free extract after expression in *E. coli* BL21-CodonPlus(DE3)-RP. The lack of any noticeable absorbance in the range between 300 and 800 nm indicates that Ldi was purified as a cofactor-free enzyme; Figure S5: An N-terminal signal sequence favors localization of Ldi in the *E. coli* periplasm. After expression, *E. coli* periplasm (P) and cytosol (C) were separated as described in the Materials and Methods section. Recombinant Ldi in isolated fractions was detected via immunoblotting. Controls were treated with ddH_2_O instead of the solutions inducing formation of spheroplasts; Figure S6: Comparison of circular dichroism (CD) spectra of Ldi wild type enzyme and variants confirms the overall integrity of single amino acid exchange variants. Ldi wild type enzyme and variants were purified in parallel. After dilution to an A_280_ of approx. 0.8, far-UV CD spectra were collected between 190 and 260 nm. Data are expressed in mean residue ellipticity [Θ] in deg cm^2^ dmol^−1^; Table S1: Codon-optimized DNA sequence for expression of C-terminally His_10_-tagged Ldi in *E. coli*; Table S2: Codon-optimized DNA sequence for expression of C-terminally His_10_-tagged OmpA-Ldi in *E. coli*; Table S3: Codon-optimized DNA sequence for expression of N-terminally His_10_-tagged nosig-Ldi in *E. coli*; Table S4: Codon-optimized DNA sequence for expression of C-terminally His_10_-tagged nosig-Ldi in *E. coli*; Table S5: Primers used for amplification of Ldi constructs for cloning into pET26b(+) vector. Underlined nucleotides are marking restriction sites; Table S6: Primers used for site-directed mutagenesis of Ldi cysteine residues; Table S7: *E. coli* strains tested for expression of Ldi.
