# Supplementary material for: Exploring Castellaniella defragrans Linalool (De)hydratase-Isomerase for Enzymatic Hydration of Alkenes
Source: Molecules. 2019 Jun 1;24(11):2092. doi: 10.3390/molecules24112092 (PMC6600392; doi:10.3390/molecules24112092)
Supplement: Supplementary file 1 [file molecules-24-02092-s001.pdf]

Supporting Information

Supporting Tables

Table S1: Codon-optimized DNA sequence for expression of C-terminally His_{10}-tagged Ldi in *E. coli*

ATGCGCTTTACCCTGAAAACCACTGCTATTGTCTCTGCTGCTGCACTGCTGGCCGGCTTCGGTCCGCCACCGCGTGCGGCG
GAACTGCCGCCAGGCCGTCTGGCCACCACCGAGGACTATTTGCGCAGCAGGCAAAACAGGCCGTTACGCCGGACGTGAT
GGCGCAATTGGCGTATATGAATTACATCGACTTTATCAGCCCGTTTTACAGCCGTGGTTGTAGCTTCGAGGCATGGGAACT
GAAGCACACGCCGCAGCGTGTTATCAAGTATAGCATTGCGTTCTATGCGTACGGTTTGGCGTCCGTTGCGCTGATTGATCC
GAAATTGCGTGCGCTGGCTGGCCACGATCTGGATATCGCCGTGAGCAAGATGAAATGCAAGCGTGTGTGGGGCGACTGGG
AAGAGGACGGTTTTGGTACGGACCCGATTGAGAAAGAAAACATTATGTACAAAGGCCACCTGAACCTGATGTATGGTCTG
TATCAGCTGGTTACTGGCAGCCGCCGTTATGAGGCGGAGCACGCGCATCTGACCCGCATCATCCATGATGAAATTGCAGCC
AACCCGTTCGCGGGCATCGTTTGCGAGCCTGATAACTACTTTGTTCAATGTAATAGCGTCGCATACCTGAGCCTGTGGGTG
TACGACCGTCTGCATGGCACGGATTACCGTGCAGCGACCCGTGCGTGGCTGGACTTCATCCAAAAAGACTTGATCGACCCG
GAGCGTGGCGCATTCTACTTGTCTTACCATCCGGAGTCCGGTGCAGTGAAACCGTGGATTAGCGCGTACACCACCGCCTGG
ACGCTGGCAATGGTCCACGGCATGGACCCGGCGTTTAGCGAGCGTTATTACCCGCGCTTCAAGCAAACCTTCGTTGAAGTG
TACGATGAAGGTCGCAAAGCGCGCGTGCGCGAAACCGCCGGTACGGATGATGCAGATGGTGGTGTCGGTCTGGCCAGCGC
CTTCACCCTGCTGCTGGCACGCGAGATGGGTGACCAGCAACTGTTTGACCAGCTGCTGAATCACCTGGAACCGCCTGCTAA
GCCGTCCATTGTCAGCGCGAGCTTGCGTTATGAGCATCCGGGTTCGCTGCTGTTCGATGAATTGCTGTTCCTGGCGAAGGT
CCACGCGGGTTTTGGTGCGCTGCTGCGTATGCCGCCACCGGCTGCCAAGCTGGCAGGCAAGCATCACCATCACCATCACCA
TCACCATCACTAATAA

Table S2: Codon-optimized DNA sequence for expression of C-terminally His_{10}-tagged OmpA-Ldi in *E. coli*

ATGAAAAAGACAGCTATCGCGATTGCAGTGGCACTGGCTGGTTTCGCTACCGTAGCGCAGGCCGCGGAACTGCCGCCAGG
CCGTCTGGCCACCACCGAGGACTATTTGCGCAGCAGGCAAAACAGGCCGTTACGCCGGACGTGATGGCGCAATTGGCGT
ATATGAATTACATCGACTTTATCAGCCCGTTTTACAGCCGTGGTTGTAGCTTCGAGGCATGGGAACTGAAGCACACGCCGC
AGCGTGTTATCAAGTATAGCATTGCGTTCTATGCGTACGGTTTGGCGTCCGTTGCGCTGATTGATCCGAAATTGCGTGCGCT
GGCTGGCCACGATCTGGATATCGCCGTGAGCAAGATGAAATGCAAGCGTGTGTGGGGCGACTGGGAAGAGGACGGTTTTG
GTACGGACCCGATTGAGAAAGAAAACATTATGTACAAAGGCCACCTGAACCTGATGTATGGTCTGTATCAGCTGGTTACT
GGCAGCCGCCGTTATGAGGCGGAGCACGCGCATCTGACCCGCATCATCCATGATGAAATTGCAGCCAACCCGTTCGCGGG
CATCGTTTGCGAGCCTGATAACTACTTTGTTCAATGTAATAGCGTCGCATACCTGAGCCTGTGGGTGTACGACCGTCTGCAT
GGCACGGATTACCGTGCAGCGACCCGTGCGTGGCTGGACTTCATCCAAAAAGACTTGATCGACCCGGAGCGTGGCGCATT
CTACTTGTCTTACCATCCGGAGTCCGGTGCAGTGAAACCGTGGATTAGCGCGTACACCACCGCCTGGACGCTGGCAATGGT
CCACGGCATGGACCCGGCGTTTAGCGAGCGTTATTACCCGCGCTTCAAGCAAACCTTCGTTGAAGTGTACGATGAAGGTCG
CAAAGCGCGCGTGCGCGAAACCGCCGGTACGGATGATGCAGATGGTGGTGTCGGTCTGGCCAGCGCCTTCACCCTGCTGC
TGGCACGCGAGATGGGTGACCAGCAACTGTTTGACCAGCTGCTGAATCACCTGGAACCGCCTGCTAAGCCGTCCATTGTCA
GCGCGAGCTTGCGTTATGAGCATCCGGGTTCGCTGCTGTTCGATGAATTGCTGTTCCTGGCGAAGGTCCACGCGGGTTTTG
GTGCGCTGCTGCGTATGCCGCCACCGGCTGCCAAGCTGGCAGGCAAGCATCACCATCACCATCACCATCACCATCACTAAT
AA

Table S3: Codon-optimized DNA sequence for expression of N-terminally His_{10}-tagged nosig-Ldi in *E. coli*

ATGCATCACCATCACCATCACCATCACCATCACGCGGAACTGCCGCCAGGCCGTCTGGCCACCACCGAGGACTATTTTGCG
CAGCAGGCAAAACAGGCCGTTACGCCGGACGTGATGGCGCAATTGGCGTATATGAATTACATCGACTTTATCAGCCCGTTT
TACAGCCGTGGTTGTAGCTTCGAGGCATGGGAACTGAAGCACACGCCGCAGCGTGTTATCAAGTATAGCATTGCGTTCTAT
GCGTACGGTTTGGCGTCCGTTGCGCTGATTGATCCGAAATTGCGTGCGCTGGCTGGCCACGATCTGGATATCGCCGTGAGC
AAGATGAAATGCAAGCGTGTGTGGGGCGACTGGGAAGAGGACGGTTTTGGTACGGACCCGATTGAGAAAGAAAACATTA
TGTACAAAGGCCACCTGAACCTGATGTATGGTCTGTATCAGCTGGTTACTGGCAGCCGCCGTTATGAGGCGGAGCACGCGC
ATCTGACCCGCATCATCCATGATGAAATTGCAGCCAACCCGTTCGCGGGCATCGTTTGCGAGCCTGATAACTACTTTGTTC
AATGTAATAGCGTCGCATACCTGAGCCTGTGGGTGTACGACCGTCTGCATGGCACGGATTACCGTGCAGCGACCCGTGCGT
GGCTGGACTTCATCCAAAAAGACTTGATCGACCCGGAGCGTGGCGCATTCTACTTGTCTTACCATCCGGAGTCCGGTGCAG
TGAAACCGTGGATTAGCGCGTACACCACCGCCTGGACGCTGGCAATGGTCCACGGCATGGACCCGGCGTTTAGCGAGCGT
TATTACCCGCGCTTCAAGCAAACCTTCGTTGAAGTGTACGATGAAGGTCGCAAAGCGCGCGTGCGCGAAACCGCCGGTAC
GGATGATGCAGATGGTGGTGTCGGTCTGGCCAGCGCCTTCACCCTGCTGCTGGCACGCGAGATGGGTGACCAGCAACTGTT
TGACCAGCTGCTGAATCACCTGGAACCGCCTGCTAAGCCGTCCATTGTCAGCGCGAGCTTGCGTTATGAGCATCCGGGTTC
GCTGCTGTTCGATGAATTGCTGTTCCTGGCGAAGGTCCACGCGGGTTTTGGTGCGCTGCTGCGTATGCCGCCACCGGCTGC
CAAGCTGGCAGGCAAGTAATAA

Table S4: Codon-optimized DNA sequence for expression of C-terminally His_{10}-tagged nosig-Ldi in *E. coli*

ATGGCGGAACTGCCGCCAGGCCGTCTGGCCACCACCGAGGACTATTTTGCGCAGCAGGCAAAACAGGCCGTTACGCCGGA
CGTGATGGCGCAATTGGCGTATATGAATTACATCGACTTTATCAGCCCGTTTTACAGCCGTGGTTGTAGCTTCGAGGCATG
GGAACTGAAGCACACGCCGCAGCGTGTTATCAAGTATAGCATTGCGTTCTATGCGTACGGTTTGGCGTCCGTTGCGCTGAT
TGATCCGAAATTGCGTGCGCTGGCTGGCCACGATCTGGATATCGCCGTGAGCAAGATGAAATGCAAGCGTGTGTGGGGCG
ACTGGGAAGAGGACGGTTTTGGTACGGACCCGATTGAGAAAGAAAACATTATGTACAAAGGCCACCTGAACCTGATGTAT
GGTCTGTATCAGCTGGTTACTGGCAGCCGCCGTTATGAGGCGGAGCACGCGCATCTGACCCGCATCATCCATGATGAAATT
GCAGCCAACCCGTTCGCGGGCATCGTTTGCGAGCCTGATAACTACTTTGTTCAATGTAATAGCGTCGCATACCTGAGCCTG
TGGGTGTACGACCGTCTGCATGGCACGGATTACCGTGCAGCGACCCGTGCGTGGCTGGACTTCATCCAAAAAGACTTGATC
GACCCGGAGCGTGGCGCATTCTACTTGTCTTACCATCCGGAGTCCGGTGCAGTGAAACCGTGGATTAGCGCGTACACCACC
GCCTGGACGCTGGCAATGGTCCACGGCATGGACCCGGCGTTTAGCGAGCGTTATTACCCGCGCTTCAAGCAAACCTTCGTT
GAAGTGTACGATGAAGGTCGCAAAGCGCGCGTGCGCGAAACCGCCGGTACGGATGATGCAGATGGTGGTGTCGGTCTGGC
CAGCGCCTTCACCCTGCTGCTGGCACGCGAGATGGGTGACCAGCAACTGTTTGACCAGCTGCTGAATCACCTGGAACCGCC
TGCTAAGCCGTCCATTGTCAGCGCGAGCTTGCGTTATGAGCATCCGGGTTCGCTGCTGTTCGATGAATTGCTGTTCCTGGCG
AAGGTCCACGCGGGTTTTGGTGCGCTGCTGCGTATGCCGCCACCGGCTGCCAAGCTGGCAGGCAAGCATCACCATCACCAT
CACCATCACCATCACTAATAA

Table S5: Primers used for amplification of Ldi constructs for cloning into pET26b(+) vector. Underlined nucleotides are marking restriction sites

Primer	Sequence (5' → 3')
Fw(Ldi_*Nde*I)	TATACATATGCGCTTTACCCTG
Rv(Ldi_His_{10}_*Hind*III)	GCCAAGCTTATTAGTGATGGTGATGGTGATGGTGATGGTGATGCTTGCCTGCCAGCTTG
Fw(OmpA-Ldi_*Nde*I)	TATACATATGAAAAAGACAGCTATC
Fw(nosig-Ldi_*Nde*I)	TATACATATGGCGGAACTGCC
Fw(His_{10}-nosig-Ldi_*Nde*I)	GCCCATATGCATCACCATCACCATCACCATCACCATCACGCGGAACTGCCGCCAG
Rv(His_{10}-nosig-Ldi_*Hind*III)	GCCAAGCTTATTACTTGCCTGCCAGCTTGGC

Table S6: Primers used for site-directed mutagenesis of Ldi cysteine residues

Primer	Sequence (5' → 3')
Fw(Ldi_C48A)	CGTTTTACAGCCGTGGTGCTAGCTTCGAGGCATGGGAAC
Rv(Ldi_C48A)	GTTCCCATGCCTCGAAGCTAGCACCACGGCTGTAAAACG
Fw(Ldi_C48S)	CGTTTTACAGCCGTGGTAGCAGCTTCGAGGCATGGGAAC
Rv(Ldi_C48S)	GTTCCCATGCCTCGAAGCTGCTACCACGGCTGTAAAACG
Fw(Ldi_C101A)	CGTGAGCAAGATGAAAGCTAAGCGTGTGTGGGGCG
Rv(Ldi_C101A)	CGCCCCACACACGCTTAGCTTTCATCTTGCTCACG
Fw(Ldi_C101S)	CGTGAGCAAGATGAAAAGCAAGCGTGTGTGGGGCG
Rv(Ldi_C101S)	CGCCCCACACACGCTTGCTTTTCATCTTGCTCACG
Fw(Ldi_C196A)	GTTCGCGGGCATCGTTGCTGAGCCTGATAACTACT
Rv(Ldi_C196A)	AGTAGTTATCAGGCTCAGCAACGATGCCCGCGAAC
Fw(Ldi_C196S)	GTTCGCGGGCATCGTTAGCGAGCCTGATAACTACT
Rv(Ldi_C196S)	AGTAGTTATCAGGCTCGCTAACGATGCCCGCGAAC
Fw(Ldi_C205A)	GATAACTACTTTGTTCAAGCTAATAGCGTCGCATACCTG
Rv(Ldi_C205A)	CAGGTATGCGACGCTATTAGCTTGAACAAAGTAGTTATC

Fw(Ldi_C205S)	GATAACTACTTTGTTCAAAGCAATAGCGTCGCATACCTG
Rv(Ldi_C205S)	CAGGTATGCGACGCTATTGCTTTGAACAAAGTAGTTATC

Table S7: *E. coli* strains tested for expression of Ldi

Strain	Notable properties
E. coli BL21-CodonPlus(DE3)-RIL	Extra copies of tRNAs genes (arginine, isoleucine, leucine)
E. coli BL21 TaKaRa 5	Co-expression of *E. coli* Trigger Factor (TF) chaperone
E. coli BL21 TaKaRa 1	Co-expression of chaperones dnaK-dnaJ-grpE-groES-groEL
E. coli Rosetta™(DE3)pLysS	Presence of rare tRNA genes on the same plasmid that carry the T7 lysozyme gene
E. coli BL21-CodonPlus(DE3)-RP	Extra copies of the tRNA genes recognizing arginine (AGA, AGG) and proline (CCC).
E. coli K12 ER2508	Knock out of major ATP-dependent proteases in *E. coli* cytosol (Δ*lon*, Δ*zjc*)
E. coli TUNER™	Lac permease (lacZY) deletion mutant. Allows for uniform entry of IPTG into all cells in the population.
E. coli ArcticExpress	Co-expression of cold-adapted chaperonins Cpn10 and Cpn60 from psychrophilic bacterium *Oleispira antartica*
E. coli LEMO23(DE3)	Precisely tunable expression by modulation of T7 lysozyme level by expression from well titrable rhamnose promoter

Table S8: β-myrcene hydration activity of Ldi cysteine variants in whole cell biotransformations with *E. coli*. 'i.a.': Inactive

Ldi variant	(*S*)-(+)-linalool production from β-myrcene / ng μL^{-1}
WT	1.03E+4
C48A	ia
C48S	ia
C101A	ia
C101S	ia
C170A	ia
C170S	ia
C179A	ia
C179S	ia

Supporting Figures

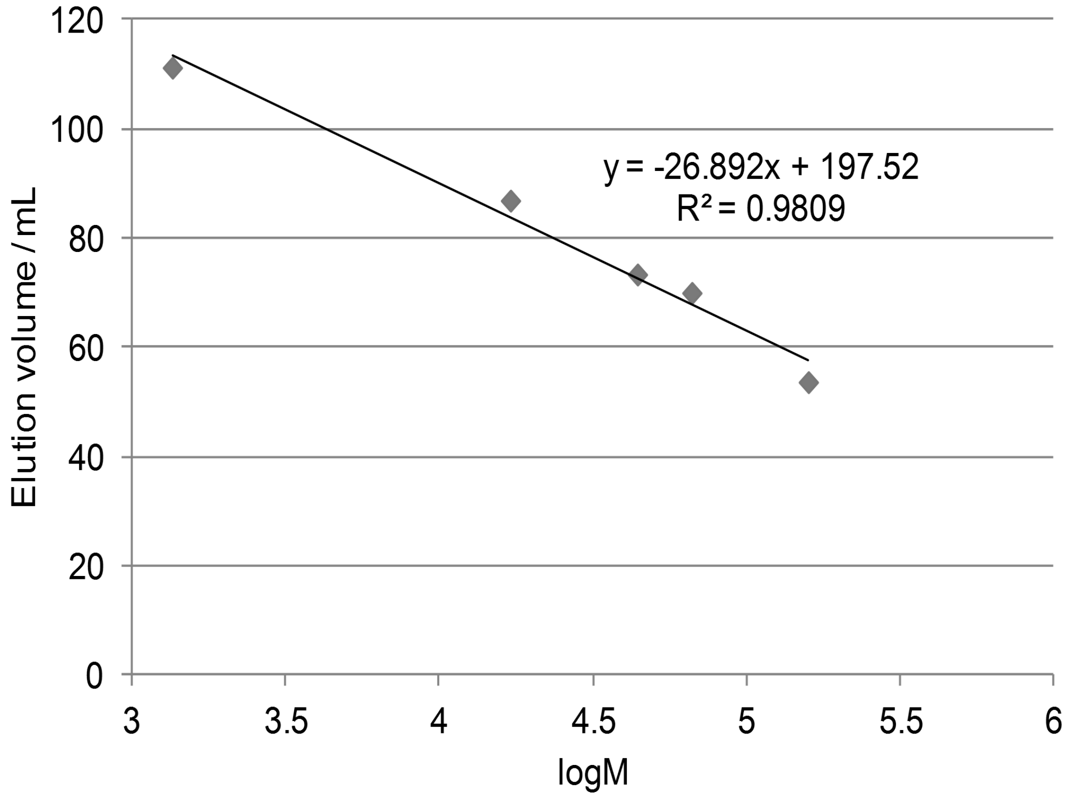

Figure S1: Gel filtration calibration curve generated with standard proteins listed in the Materials and Methods section.

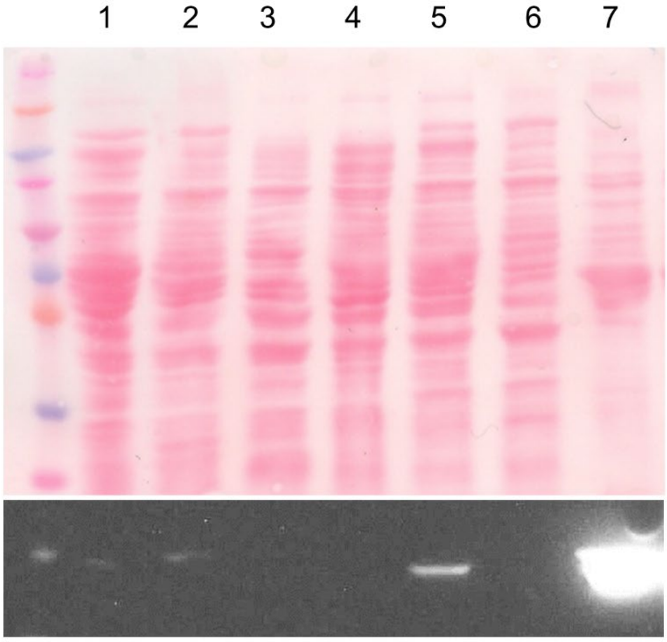

	Strain	Plasmid
1	*E. coli* LEMO21(DE3)	pMS470_Ldi
2	*E. coli* LEMO21(DE3)	pMS470 DsbC_Ldi
3	*E. coli* Origami	pMS470_Ldi
4	*E. coli* Origami	pMS470 DsbC_Ldi
5	*E. coli* BL21star(DE3)	pMS470_Ldi
6	*E. coli* BL21star(DE3)	pMS470 DsbC_Ldi
7	*E. coli* BL21star(DE3), insoluble fraction	pMS470_Ldi

Figure S2: Initial expression studies of Ldi in different *E. coli* strains indicated poor expression of soluble protein. Ldi was expressed from a pMS470 vector with and without co-expression of truncated DsbC. The level of Ldi in *E. coli* lysate was analyzed by immunoblotting.

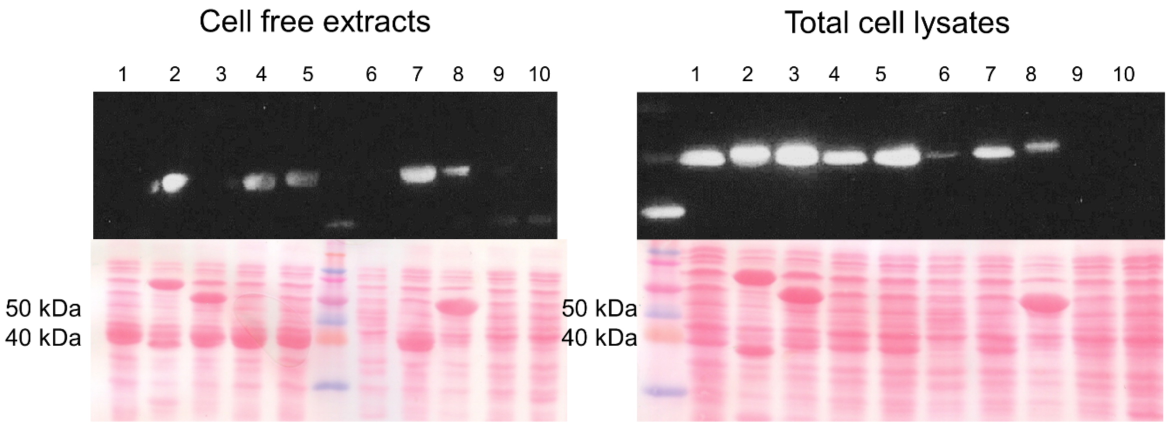

	Strain	Plasmid
1	*E. coli* BL21-CodonPlus(DE3)-RIL	pET26b(+)_Ldi-His$_{10}$
2	*E. coli* BL21 TaKaRa 5	pET26b(+)_Ldi-His$_{10}$
3	*E. coli* BL21 TaKaRa 1	pET26b(+)_Ldi-His$_{10}$
4	*E. coli* Rosetta^{TM}(DE3)pLysS	pET26b(+)_Ldi-His$_{10}$
5	*E. coli* BL21-CodonPlus(DE3)-RP	pET26b(+)_Ldi-His$_{10}$
6	*E. coli* K12 ER2508	pET26b(+)_Ldi-His$_{10}$
7	*E. coli* TUNER^{TM}	pET26b(+)_Ldi-His$_{10}$
8	*E. coli* ArcticExpress	pET26b(+)_Ldi-His$_{10}$
9	*E. coli* LEMO23(DE3)	pET26b(+)_Ldi-His$_{10}$
10	*E. coli* BL21star	pET26b(+)

Figure S3: Expression of Ldi in different *E. coli* strains increases the yield of soluble protein in specific set ups. Nine different *E. coli* strains were tested for expression of Ldi, and after harvesting the cells, His$_{10}$-tagged Ldi was detected in total cell lysates and cell free extracts by immunoblotting.

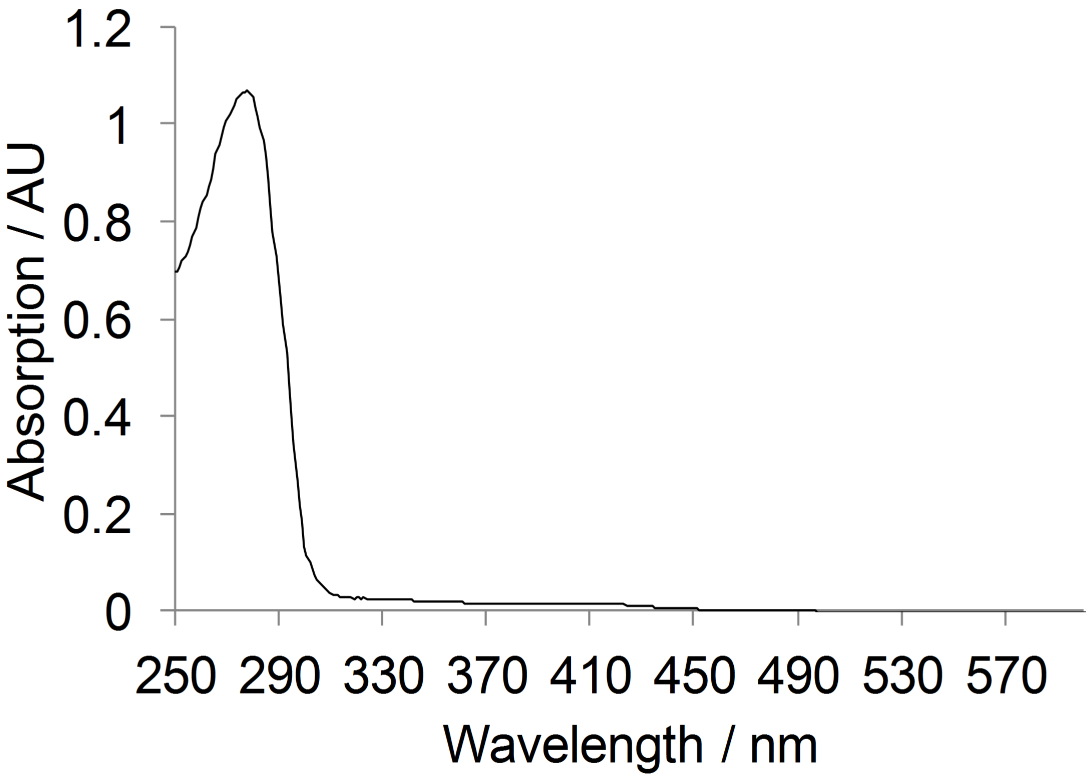

Figure S4: UV-Vis absorption spectrum of purified Ldi. The enzyme was His_{10}-tag purified from cell free extract after expression in *E. coli* BL21-CodonPlus(DE3)-RP. The lack of any noticeable absorbance in the range between 300 and 800 nm indicates that Ldi was purified as a cofactor-free enzyme.

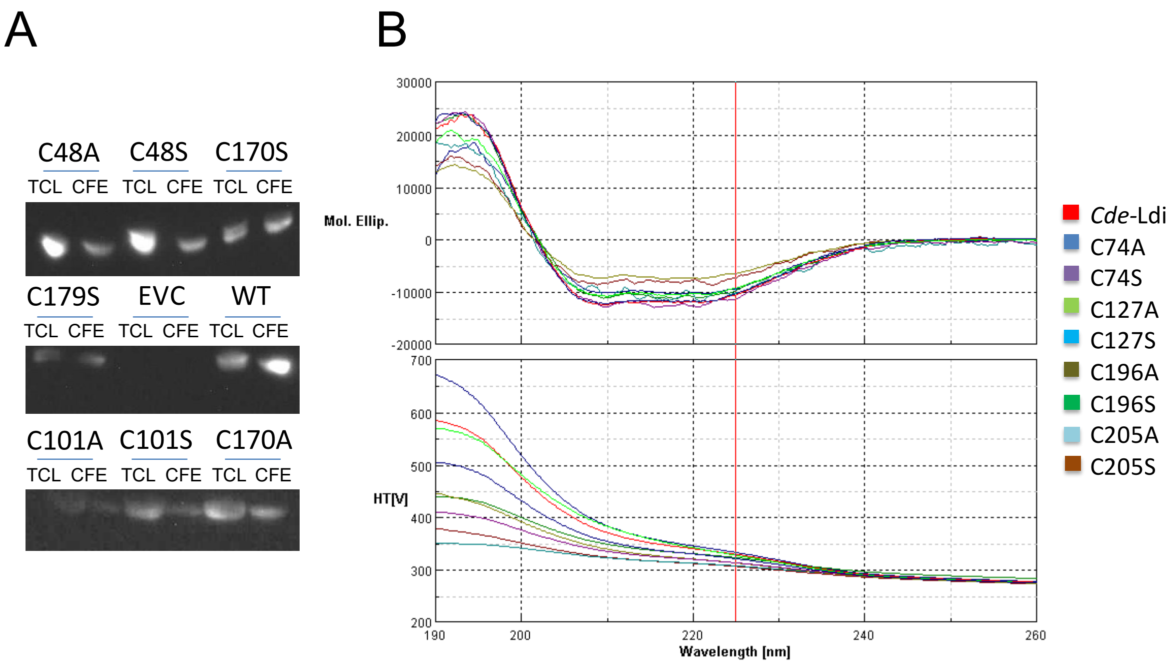

Figure S5: Expression and overall structure of Ldi cysteine variants. (A) Expression of cysteine variants and Ldi wild type (WT) in total cell lysates (TCL) and cell free extracts (CFE) was confirmed by immunoblotting. EVC: Empty vector control. (B) Comparison of circular dichroism (CD) spectra of Ldi wild type enzyme and variants confirms the overall integrity of single amino acid exchange variants. Ldi wild type enzyme and variants were purified in parallel. After dilution to an A_{280} of approx. 0.8, far-UV CD spectra were collected between 190 and 260 nm. Data are expressed in mean residue ellipticity [Θ] in deg cm^2 $dmol^{-1}$.

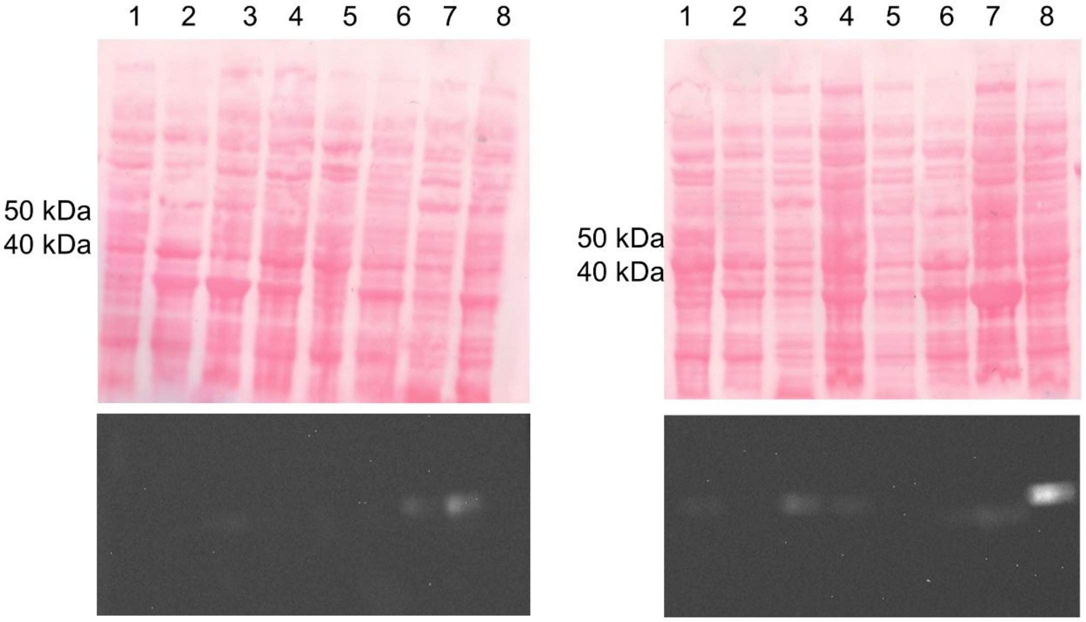

Sample		
1	EVC	P, control
2		C, control
3		P, isolated
4		C, isolated
5	Ldi	P, control
6		C, control
7		P, isolated
8		C, isolated

Sample		
1	OmpA-Ldi	P, control
2		C, control
3		P, isolated
4		C, isolated
5	His_{10}-nosig-Ldi	P, control
6		C, control
7		P, isolated
8		C, isolated

Figure S6: An N-terminal signal sequence favors localization of Ldi in the *E. coli* periplasm. After expression, *E. coli* periplasm (P) and cytosol (C) were separated as described in the Materials and Methods section. Recombinant Ldi in isolated fractions was detected via immunoblotting. Controls were treated with ddH_2O instead of the solutions inducing formation of spheroplasts.
